# Effects of Extended-Release Cornstarch Supplementation on Glycemic Stability and Metabolic Parameters in Korean Patients with Glycogen Storage Disease

**DOI:** 10.3390/nu18071094

**Published:** 2026-03-29

**Authors:** Jungyun Han, Minjy Kim, Na Yeon Lee, Yunkoo Kang

**Affiliations:** 1 Department of Medicine, Yonsei University Wonju College of Medicine, Wonju 26426, Republic of Korea; 2020291008@yonsei.ac.kr (J.H.); minjy4862@yonsei.ac.kr (M.K.); nayeonlee0213@yonsei.ac.kr (N.Y.L.); 2 Department of Pediatrics, Yonsei University Wonju College of Medicine, Wonju 26426, Republic of Korea

**Keywords:** glycogen storage disease, Glycosade, uncooked cornstarch, continuous glucose monitoring, glycemic variability, metabolic parameters

## Abstract

**Background/Objectives:** Patients with hepatic glycogen storage disease (GSD) require frequent nighttime intake of uncooked corn starch (UCCS) to prevent fasting hypoglycemia, which imposes a substantial burden. Glycosade, an extended-release cornstarch, was developed to prolong overnight glucose availability. However, data regarding South Korean patients are limited. Therefore, we aimed to evaluate the efficacy and safety of Glycosade in South Korean patients with hepatic GSD. **Methods:** In this single-center prospective observational study, patients with hepatic GSD underwent laboratory evaluations before and 1 month after Glycosade administration. Continuous glucose monitoring (CGM) was performed during UCCS and Glycosade administration periods. The nocturnal mean glucose, coefficient of variation, time in range (70–180 mg/dL), and time below the range (<70 and <54 mg/dL) were compared between the periods using paired analyses. **Results:** No significant differences were observed in the nocturnal CGM metrics between the treatment periods. However, time-aligned CGM profiles revealed distinct temporal patterns, with a decline in glucose levels approximately 3–4 h after UCCS intake, whereas Glycosade showed a more sustained glucose profile over an extended period. Liver enzyme and lipid levels improved significantly after 1 month of Glycosade supplementation. **Conclusions:** In a cohort of South Korean patients with hepatic GSD, Glycosade maintained nocturnal glycemic stability comparable to that of conventional cornstarch without increasing the risk of hypoglycemia. Glycosade was also associated with improved biochemical parameters, supporting its role in nighttime dietary management.

## 1. Introduction

Glycogen storage disease (GSD) is an inherited pan-ethnic metabolic disorder caused by enzymatic defects that result in the abnormal storage and breakdown of glycogen [1]. Glycogen is primarily stored in the liver and skeletal muscle; hepatic glycogen maintains euglycemia during fasting, whereas muscle glycogen supplies energy during high-intensity exercise [2]. Clinically, GSD is classified based on the underlying enzymatic defect, predominant organ involvement (liver or muscle), and associated phenotypic manifestations.

Among the hepatic GSDs in pediatric patients, the most prevalent subtypes include GSD type I (glucose-6-phosphatase deficiency), type III (glycogen debranching enzyme deficiency), and type IX (phosphorylase kinase deficiency). Although disease severity varies, the hallmark features of hepatic involvement and fasting hypoglycemia are universal among the subtypes. In GSD type I, impaired glycogenolysis and gluconeogenesis limit endogenous glucose production, resulting in severe hypoglycemia and secondary metabolic abnormalities such as hyperuricemia, hypertriglyceridemia, and lactic acidosis [1]. In contrast, gluconeogenesis and fatty acid oxidation remain relatively preserved in GSD types III and IX. Thus, these two types are characterized by comparatively milder hypoglycemia; however, the resulting compensatory increases in fatty acid β-oxidation may promote ketosis [3].

The current management of hepatic GSD centers on the strict prevention of fasting hypoglycemia and mitigation of downstream metabolic complications to preserve hepatic function and improve long-term outcomes. As of the early 1980s, uncooked cornstarch (UCCS) has been a cornerstone of therapy; the slow intestinal digestion/absorption of UCCS provides sustained release of glucose, thereby reducing hypoglycemic events [4]. Standard dietary regimens typically involve the distribution of complex carbohydrates evenly over 24 h, with nighttime coverage considered essential to prevent nocturnal hypoglycemia. Common dosing strategies include approximately 1.6 g/kg of UCCS every 3–4 h in young children and 1.7–2.5 g/kg every 4–5 h in adolescents and adults, accompanied by blood glucose and lactate level monitoring [5].

Despite its clinical benefits, UCCS has a relatively short duration of action and requires additional overnight doses. This requirement contributes to chronic sleep fragmentation and imposes a substantial psychological and caregiving burden, ultimately impairing the quality of life of the patients and their families. Moreover, missed nighttime doses can precipitate acute hypoglycemia and morning lactic acidosis.

To address the limitations of conventional UCCS therapy, Glycosade, a modified high amylopectin starch designed for extended glucose release, was developed to prolong fasting tolerance. Glycosade maintains euglycemia for >7 h by slowing gastrointestinal digestion/absorption, thereby reducing or eliminating the need for nighttime starch intake [6]. In a double-blind randomized crossover trial, Correia et al. showed improved overnight glucose stability in patients using Glycosade compared with that in patients using UCCS [6]. Long-term follow-up data from Western cohorts have also suggested sustained metabolic control and improved sleep-related outcomes [7].

In the modern management of GSD, continuous glucose monitoring (CGM) has emerged as an indispensable strategy for overcoming the limitations of intermittent monitoring. CGM is particularly valuable for detecting asymptomatic and nocturnal hypoglycemia, which are common challenges that often evade detection using standard self-blood glucose monitoring [8,9]. Furthermore, CGM metrics facilitate the precise titration of dietary therapies such as Glycosade administration by providing detailed glycemic profiles. This enables clinicians to minimize iatrogenic overtreatment and optimize metabolic control while reducing the burden of frequent finger-stick testing [10]. Consequently, CGM is increasingly recognized as a standard modality for evaluating the safety and efficacy of therapeutic modifications.

However, most of the clinical evidence regarding Glycosade has been derived from Western populations, and data on its efficacy in Asian patients, who may have distinct dietary patterns and genetic backgrounds, remain limited. With the recent expansion of national coverage for Glycosade in South Korea, its integration into routine dietary management is expected to increase, amplifying the need for evidence of its impact in South Korean patients. Therefore, in this study, we aimed to evaluate the efficacy and safety of Glycosade in South Korean patients with hepatic GSD, using CGM-derived metrics as the primary outcomes.

## 2. Methods

### 2.1. Study Design

**Figure 1:**
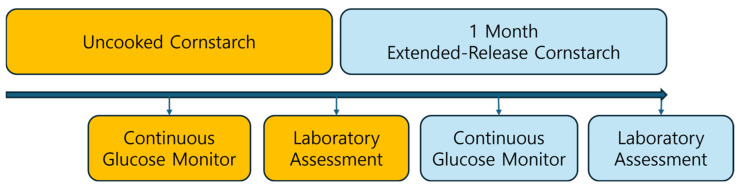
Study design: baseline assessment data during the period of uncooked cornstarch therapy were compared with follow-up assessment data after 1 month of therapy using extended-release cornstarch (Glycosade); CGM and laboratory assessment data were analyzed.

This was a single-center, prospective, before-and-after observational study in which we evaluated the metabolic changes following Glycosade^®^ (Vitaflo International Ltd., Liverpool, UK) supplementation in a cohort of patients with GSD. Baseline laboratory evaluations were performed before Glycosade administration and repeated at 1 month after Glycosade use was initiated (Figure 1). This study was not a clinical trial; rather, this was a prospective observational study evaluating real-world metabolic changes after Glycosade supplementation. No formal sample size calculation was performed due to the exploratory nature of the study and the limited number of eligible patients with hepatic GSD in a single-center setting.

### 2.2. Participants

Patients with a confirmed diagnosis of GSD who were followed up with at Wonju Severance Christian Hospital (Wonju-si, Gangwon-do, Republic of Korea) were eligible for inclusion. Both pediatric and adult patients were included in this study. A total of 22 patients were enrolled in the study.

### 2.3. Intervention

All 22 participants had received standard cornstarch-based dietary therapy before study enrollment. Glycosade was administered as a nighttime cornstarch replacement according to individual clinical requirements. The patients consumed Glycosade for 1 month without changing other aspects of their routine dietary management. Nighttime Glycosade doses were adjusted individually according to CGM trends and clinical assessment of nocturnal glucose stability. In general, downward nocturnal glucose trends or hypoglycemia prompted dose escalation, whereas stable overnight glucose profiles or hyperglycemia prompted dose maintenance or reduction, with consideration of clinical symptoms and morning glucose patterns.

No other medication changes or dietary interventions were introduced during the study period.

### 2.4. Continuous Glucose Monitoring

CGM data were obtained during two treatment periods: one in which the patients were using conventional cornstarch therapy and one in which patients were using Glycosade. CGM was performed during each period, and paired CGM datasets were analyzed for each patient. Nighttime periods were defined individually based on CGM timestamps to reflect patient-specific feeding schedules. For the time-aligned CGM analysis, glucose values were re-aligned based on the timing of nighttime starch intake (time 0), and data were analyzed over a fixed 6-h window following intake. This approach differs from conventional clock-time-based analysis and was applied to better capture the temporal glucose response to starch ingestion.

Glycemic variability was assessed using the coefficient of variation (CV, %). Nocturnal glycemic control was evaluated by analyzing the nighttime mean glucose levels, time in range (TIR) of 70–180 mg/dL (%), time below range (TBR) < 70 mg/dL (%), and TBR < 54 mg/dL (%). Nighttime was defined as the sleep period, according to the CGM recording protocol. These measures were compared between the conventional cornstarch and Glycosade periods for each patient.

Therapeutic efficacy was evaluated using CGM-derived nocturnal glycemic metrics, including mean glucose, coefficient of variation, time in range, and time below range, together with changes in biochemical parameters after 1 month of Glycosade supplementation. Safety was assessed based on the occurrence of clinically relevant nocturnal hypoglycemia during CGM and the absence of reported serious adverse events during the study period.

CGM data were obtained using the Dexcom G7 continuous glucose monitoring system (Dexcom, Inc., San Diego, CA, USA). Data were integrated and analyzed using the PASTA digital health platform (Kakao Healthcare Corp., Seongnam, Republic of Korea).

### 2.5. Data Collection

Laboratory data obtained during the first study visit before and after 1 month of Glycosade supplementation were extracted from electronic medical records. Laboratory parameters included aspartate aminotransferase (AST), alanine aminotransferase (ALT), triglyceride, total cholesterol, and creatine kinase levels.

CGM data were obtained using the PASTA digital health platform, which integrates glucose data directly from connected CGM devices into standardized datasets for analysis. All participating patients were equipped with the Dexcom G7 continuous glucose monitoring system.

TBR was defined as the percentage of time in which the glucose levels fell below 70 mg/dL and below 54 mg/dL.

### 2.6. Statistical Analysis

Continuous variables are summarized as medians with interquartile ranges (IQRs) owing to the non-normal data distribution. Categorical variables are presented as counts and percentages. Comparisons before and after Glycosade administration were performed using the Wilcoxon signed-rank test for paired data. This test was chosen owing to the non-normal variable distribution. All statistical analyses were conducted using R software (version 4.3.2; R Foundation for Statistical Computing, Vienna, Austria). A two-sided *p*-value < 0.05 was considered statistically significant where applicable.

### 2.7. Ethical Approval

This study was approved by the Institutional Review Board of Wonju Severance Christian Hospital (IRB No. CR325019). Written informed consent was obtained from all participants or their legal guardians.

## 3. Results

### 3.1. Baseline Characteristics

The baseline characteristics of the study population are summarized in Table 1. A total of 22 patients with GSD were included in this analysis. The median age was 13 (IQR: 11–14) years, and 14 patients (63.6%) were male. The median height and weight were 149.1 (IQR: 142.5–160.9) cm and 47.1 (IQR: 41.8–57.4) kg, respectively, with a median body mass index of 21.4 (IQR: 20.4–22.8) kg/m^2^. Prior to Glycosade initiation, the median nighttime cornstarch intake was 98.0 (IQR: 83.0–101.8) g per dose. Baseline laboratory findings obtained before Glycosade administration showed the following median levels: AST, 26.0 (IQR: 22.2–29.0) U/L; ALT, 19.5 (IQR: 17.0–36.0) U/L; triglycerides, 209.5 (IQR: 147.5–359.0) mg/dL; total cholesterol, 176.0 (IQR: 157.8–211.2) mg/dL; and creatine kinase, 120.5 (IQR: 94.0–151.8) U/L.

### 3.2. Laboratory Parameters

One month after the patients initiated Glycosade administration, significant improvements were observed in the liver enzyme levels and lipid profiles (Table 2). The median AST and ALT levels decreased significantly, accompanied by decreases in triglyceride and total cholesterol levels. The creatine kinase levels also showed a modest but statistically significant decrease.

### 3.3. Cornstarch Dose Subgroup Analysis

The analysis of the nighttime starch dose according to the GSD subtype revealed that the patients with GSD Ia (*n* = 17) received a median nighttime dose of 100.0 (93.0–104.0) g during UCCS therapy and 95.0 (85.0–105.0) g during Glycosade therapy (Table 3). Although the median dose decreased, the difference was not statistically significant (*p* = 0.060).

In the patients with GSD IXa (*n* = 4), the median nighttime dose decreased from 69.0 (57.2–83.8) g with UCCS to 52.5 (45.0–60.0) g with Glycosade; however, this decrease was also not statistically significant (*p* = 0.125).

An overall decrease in the nighttime starch requirements was observed following the transition to Glycosade across the patient subtypes; however, the subgroup analyses did not show statistically significant differences.

### 3.4. CGM Outcome and Safety

No significant differences were observed in the nocturnal mean glucose, glycemic variability, or time between the conventional cornstarch (UCCS) and Glycosade periods (Table 4). No patients reported gastrointestinal intolerance, poor palatability, or other adverse effects requiring discontinuation during the Glycosade period.

### 3.5. Nocturnal CGM Analysis

**Figure 2:**
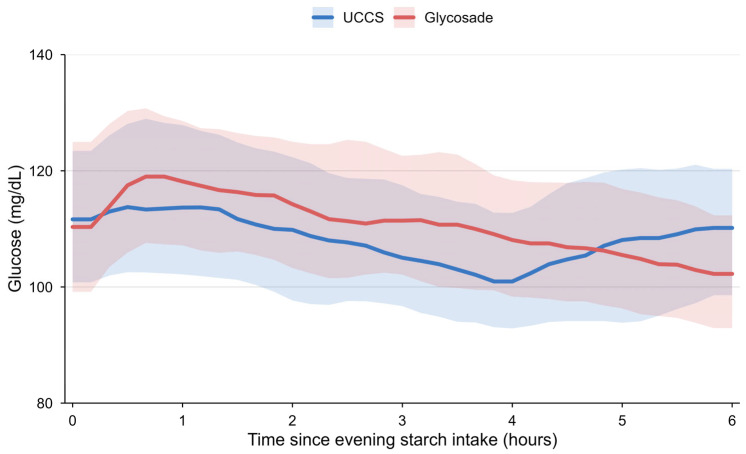
Time-aligned nocturnal CGM glucose profiles over a 6-h period following nighttime starch intake. Median glucose levels with interquartile ranges are shown. A gradual decline in glucose levels is observed approximately 3–4 h after UCCS intake, whereas Glycosade demonstrates a more sustained glucose profile over time.

The time-aligned nocturnal CGM glucose profiles are shown in Figure 2.

When aligned from the start of the nighttime starch intake, distinct temporal patterns emerged between the two treatment periods. During the UCCS period, glucose levels showed a gradual decline beginning approximately 3–4 h after intake, followed by a further downward trend toward the later time points. In contrast, during the Glycosade period, glucose levels were maintained more consistently throughout the extended observation window of up to 6 h. In addition, glucose levels immediately following Glycosade intake tended to be slightly higher compared with those during the UCCS period.

Although the overall median glucose levels and interquartile ranges overlapped between the two periods, the temporal profiles suggest that Glycosade provided a more sustained glucose release, resulting in a delayed decline in glucose levels compared with UCCS. These findings were descriptive in nature and were not subjected to formal time-series statistical testing.

## 4. Discussion

The need for frequent nighttime corn starch intake is a major burden on patients with hepatic GSD and their caregivers. To prevent the occurrence of fasting hypoglycemia, patients are often required to wake up multiple times during the night for starch administration, resulting in chronic sleep disruption, patient/caregiver fatigue, and reduced quality of life. Missed nighttime doses may lead to acute hypoglycemia or early morning metabolic decompensation, highlighting the clinical importance of stable overnight glucose coverage.

In this prospective observational study, we evaluated the impact of Glycosade, a modified extended-release cornstarch, on nocturnal glycemic control in a cohort of South Korean patients with hepatic GSD using CGM. Our results demonstrated that the nocturnal mean glucose levels, glycemic variability, and TIR remained comparable between the UCCS and Glycosade periods. Importantly, the time spent below this range remained minimal in both periods, indicating preserved nocturnal glycemic safety following Glycosade administration.

Although previous studies have reported that Glycosade prolongs fasting tolerance, most of the available data have been derived from Western populations. Dietary patterns, starch compositions, and feeding practices differ substantially across regions, and the applicability of these findings to Asian patients remains uncertain. Our findings in this South Korean cohort suggest that Glycosade may help maintain nocturnal glucose stability comparable to that achieved with conventional cornstarch therapy. These results are consistent with the findings of the GLYDE study, a large-scale international multicenter trial that demonstrated the efficacy, tolerance, and maintenance of Glycosade metabolic control across diverse GSD populations [11].

Furthermore, the present data corroborate the results of a study by Hsu et al., in which substituting bedtime cornstarch with extended-release cornstarch increased morning glucose concentrations, lowered hepatic transaminases, and improved sleep duration in a cohort of Taiwanese patients with GSD Ia [12]. The previous studies showing early crossover fasting hypoglycemia and prolonged fasting tolerance [6,13] and those including long-term observational data demonstrating preserved biochemical stability [7] collectively suggest that Glycosade may be a useful option for nighttime dietary management in patients with hepatic GSD.

In the time-aligned nocturnal CGM curves (Figure 2), a distinct temporal pattern was observed between the two starch types. During the UCCS period, glucose levels showed a gradual decline beginning approximately 3–4 h after nighttime intake, which is consistent with the known pharmacokinetic properties of conventional cornstarch that typically provides glucose coverage for about 4 h. Beyond this period, glucose levels tend to decline without additional dosing, explaining the common need for repeated nighttime administration and the associated burden on patients and caregivers.

In contrast, during the Glycosade period, glucose levels were maintained more consistently over an extended time window, suggesting a prolonged glucose release profile. Notably, glucose levels immediately following Glycosade intake tended to be slightly higher compared with those during the UCCS period, likely reflecting differences in starch composition and dosing characteristics.

Although the overall nocturnal mean glucose levels and CGM summary metrics were comparable between the two treatment periods, these time-aligned glucose patterns suggest that Glycosade may provide extended overnight glucose coverage with a different early post-intake glycemic profile.

These temporal observations were descriptive in nature and were not subjected to formal time-series statistical testing. Importantly, Glycosade was well tolerated in this cohort, with no reported serious adverse events, clinically significant hypoglycemia, or treatment-limiting side effects such as gastrointestinal intolerance. This supports the short-term safety of Glycosade in real-world clinical practice.

Although the overall nocturnal CGM summary metrics did not differ significantly between the two periods, the temporal glucose profile indicated improved physiological stability during the extended fasting period. This dissociation between the glycemic metrics and laboratory improvements may reflect reduced nocturnal metabolic stress rather than actual changes in average glucose levels. Although the reduction in the nighttime starch dose did not reach statistical significance in the subtype analyses (Table 3), most of the patients required a lower dose of Glycosade than that of conventional corn starch. Notably, these modest dose reductions were accompanied by significant improvements in the liver enzymes and lipid parameters. More stable overnight glucose availability without repeated hypoglycemic episodes or abrupt glucose fluctuations may ultimately contribute to improved hepatic and metabolic homeostasis.

Importantly, the magnitude and pattern of biochemical improvement observed in our cohort differed from those reported in previous studies using extended-release cornstarch. In adult crossover trials and longitudinal cohorts including pediatric participants, the use of extended-release starch has reliably prolonged fasting duration and reduced the nighttime treatment burden [6,7,11]. However, according to other studies, the metabolic parameters such as triglycerides, cholesterol, and uric acid generally remained stable rather than showing pronounced improvement [7,12,13]. In a recent study out of Taiwan involving extended-release corn starch at bedtime and regular corn starch in the daytime, reductions in the hepatic transaminase levels were not accompanied by significant changes in the lipid profiles [12]. In contrast, the patients in the present study exhibited concurrent improvements in the hepatic enzyme levels and atherogenic lipid parameters despite only exhibiting modest changes in the CGM-derived indices. This suggests that, in a well-controlled metabolic setting, the use of Glycosade may contribute to short-term improvements in hepatic and lipid parameters. However, the differences in baseline control, diet, and follow-up duration across these previous studies warrant cautious interpretation of the findings.

From a clinical perspective, maintaining nocturnal glycemic safety without deteriorating CGM-derived metrics is highly meaningful. Even in the absence of statistically significant improvements in the CGM indices, maintaining equivalent nocturnal glucose control while potentially reducing the frequency of nighttime feeding represents a substantial benefit for patients and caregivers. Reducing nighttime interventions may improve sleep quality, adherence, overall quality of life, and physiological outcomes that are not fully captured using glycemic metrics alone. The improvement in liver enzymes and lipid parameters may reflect reduced nocturnal metabolic stress owing to more stable overnight glucose availability.

Despite its strengths, this study has several limitations. First, the sample size was small, reflecting the rarity of hepatic GSD, and the study was exploratory in nature; therefore, it was not powered to detect small between-period differences in CGM metrics or to support robust subtype analyses. Second, this was a single-center, non-randomized before-and-after observational study without a parallel control group, and potential confounding factors such as time effects and changes in adherence cannot be completely excluded. Third, the observation period was relatively short, and longer follow-up is needed to determine the durability of the biochemical improvements and long-term safety. Fourth, the temporal patterns shown in the time-aligned nocturnal CGM curves were descriptive and were not subjected to formal time-series statistical testing; therefore, interpretations regarding prolonged glucose stability beyond specific time points should be made cautiously. Fifth, subtype-specific biochemical responses could not be robustly assessed because of the small sample size and imbalanced subtype distribution. Finally, patient-reported outcomes such as sleep quality, caregiver burden, and quality of life were not evaluated; therefore, any quality-of-life benefit remains speculative in the present study.

Despite these limitations, this study provides clinically meaningful evidence supporting the use of Glycosade in Asian patients with hepatic GSD. By demonstrating preserved nocturnal glycemic safety together with improvements in biochemical parameters, our findings suggest that extended-release cornstarch may offer practical metabolic benefits and may reduce aspects of treatment burden beyond those of conventional cornstarch therapy. Because this was a small, single-center, non-randomized before-and-after study, the observed biochemical improvements should be interpreted as hypothesis-generating rather than confirmatory. In particular, causal inference regarding the effects of Glycosade on liver enzymes and lipid parameters remains limited. Larger multicenter studies with longer follow-up periods are warranted to confirm these observations and further define the patient subgroups that may derive the greatest benefit from this therapy.

## 5. Conclusions

In this small prospective observational cohort of South Korean patients with hepatic GSD, Glycosade was associated with nocturnal glycemic stability comparable to that observed with conventional uncooked cornstarch, without an apparent increase in CGM-detected hypoglycemia. Short-term improvements in biochemical parameters were also observed; however, these findings should be interpreted cautiously given the study design and short follow-up. Glycosade may be a reasonable nighttime starch alternative in selected patients requiring stable overnight glucose control.

## Figures and Tables

**Table 1:** Baseline characteristics of participating patients with glycogen storage disease (*n* = 22).

Variables	Value (*n* = 22)
Age, years	13.0 (11.0–14.0)
Sex, male, *n* (%)	14 (63.6)
Height (cm)	149.1 (142.5–160.9)
Weight (kg)	47.1 (41.8–57.4)
Body mass index (BMI, kg/m^2^)	21.4 (20.4–22.8)
Nighttime cornstarch intake (g)	98.0 (83.0–101.8)
Aspartate aminotransferase (U/L)	26.0 (22.2–29.0)
Alanine aminotransferase (U/L)	19.5 (17.0–36.0)
Triglycerides (mg/dL)	209.5 (147.5–359.0)
Total cholesterol (mg/dL)	176.0 (157.8–211.2)
Creatine kinase (U/L)	120.5 (94.0–151.8)

Data are presented as the median (interquartile range) or number (%). Baseline laboratory values were obtained before the initiation of Glycosade therapy. Body mass index (BMI) was calculated as weight in kilograms divided by height in meters squared.

**Table 2:** Comparison of laboratory parameters before and after Glycosade administration.

Variable	UCCS	Glycosade	*p*-Value
AST (U/L)	26.0 (22.2–29.0)	20.5 (19.2–32.0)	0.004
ALT (U/L)	19.5 (17.0–36.0)	17.5 (14.0–27.2)	0.049
Triglycerides (mg/dL)	209.5 (147.5–359.0)	157.0 (129.2–319.5)	0.030
Total cholesterol (mg/dL)	176.0 (157.8–211.2)	169.0 (159.5–197.0)	0.048
Creatine kinase (U/L)	120.5 (94.0–151.8)	112.5 (87.8–135.8)	0.023

Data are presented as the median (interquartile range). The *p*-values were calculated using the Wilcoxon signed-rank test for paired comparisons as the variables were not normally distributed. AST: Aspartate aminotransferase, ALT: Alanine aminotransferase.

**Table 3:** Comparison of nighttime starch dose between UCCS and Glycosade by GSD subtype.

GSD Type	*n*	UCCS (g)	Glycosade (g)	*p*-Value
Ia	17	100.0 (93.0–104.0)	95.0 (85.0–105.0)	0.060
IXa	4	69.0 (57.2–83.8)	52.5 (45.0–60.0)	0.125
VI	1	98.0	90.0	-

Values are presented as the median (interquartile range). The *p*-values were calculated using the Wilcoxon signed-rank test for paired comparisons between UCCS and Glycosade. Statistical analyses were not performed for the GSD VI subgroup as this group comprised only one patient. UCCS: Uncooked cornstarch.

**Table 4:** Nocturnal CGM metrics before and after Glycosade therapy.

Variable	UCCS	Glycosade	*p*-Value
Mean glucose (mg/dL)	109.3 (103.3–114.0)	108.5 (104.9–116.3)	0.824
CV (%)	14.0 (12.4–15.2)	13.5 (12.2–15.5)	0.566
TIR 70–180 mg/dL (%)	99.6 (98.4–100.0)	99.1 (98.5–99.8)	0.483
TBR < 70 mg/dL (%)	0.3 (0.0–1.6)	0.7 (0.0–1.5)	0.476
TBR < 54 mg/dL (%)	0.0 (0.0–0.5)	0.0 (0.0–0.7)	0.534

The *p*-values were calculated using the Wilcoxon signed-rank test for paired comparisons. CV: Coefficient of variation, TIR: Time in range (70–180 mg/dL), TBR: Time below range (<70 or <54 mg/dL).

## Data Availability

The data supporting the findings of this study are not publicly available due to ethical and privacy restrictions and cannot be shared.

## Abbreviations

The following abbreviations are used in this manuscript:

GSD	Glycogen storage disease
UCCS	Uncooked cornstarch
CGM	Continuous glucose monitoring
CV	Coefficient of variation
TIR	Time in range
TBR	Time below range
AST	Aspartate aminotransferase
ALT	Alanine aminotransferase
IQR	Interquartile range
BMI	Body mass index
IRB	Institutional review board
